# Узловой токсический зоб у детей: особенности клинической картины, морфологические варианты

**DOI:** 10.14341/probl12738

**Published:** 2021-04-08

**Authors:** Т. Е. Иванникова, О. Б. Безлепкина, Ф. М. Абдулхабирова, А. Ю. Абросимов, М. В. Дегтярев, Н. А. Зубкова

**Affiliations:** Национальный медицинский исследовательский центр эндокринологии; Национальный медицинский исследовательский центр эндокринологии; Национальный медицинский исследовательский центр эндокринологии; Национальный медицинский исследовательский центр эндокринологии; Национальный медицинский исследовательский центр эндокринологии; Национальный медицинский исследовательский центр эндокринологии

**Keywords:** токсический узловой зоб, гипертиреоз, функциональная автономия, щитовидная железа

## Abstract

ОБОСНОВАНИЕ. Токсический узловой зоб (ТУЗ) — редкое заболевание, при котором причиной гипертиреоза является наличие узла или узлов, автономно секретирующих гормоны щитовидной железы. У детей и подростков данное состояние встречается крайне редко — в 5–7,5% всех случаев узлового зоба. Терапия ТУЗ направлена на купирование симптомов гипертиреоза с учетом злокачественного потенциала узлового образования. В доступной литературе отсутствуют данные о клиническом течении, сравнительных результатах цитологических и гистологических данных пациентов с ТУЗ, дебютировавшим в детском возрасте.ЦЕЛЬ. Анализ особенностей клинического течения, сравнение результатов цитологического и гистологического исследований ТУЗ у детей и подростков.МАТЕРИАЛЫ И МЕТОДЫ. Ретроспективное одноцентровое исследование 21 пациента с одноузловым токсическим зобом, госпитализированных в ФГБУ «НМИЦ эндокринологии» Минздрава России в период с января 2016 г. по декабрь 2019 г.РЕЗУЛЬТАТЫ. Средний возраст на момент обследования составлял 13,9 года. У 13 пациентов (65%) отмечался манифестный тиреотоксикоз, у 7 (35%) — субклинический гипертиреоз. Больше половины детей — 57,1% (n=12) не получали тиреостатической терапии. Цитологическая картина у 11 пациентов (61,1%) соответствовала доброкачественным изменениям (узловой коллоидный зоб или аденоматозный зоб) — Bethesda II, у 4 пациентов — фолликулярной опухоли — Bethesda IV, у 4 детей исследование оказалось неинформативным. 19 пациентам (90,5%) было проведено хирургическое лечение (гемитиреоидэктомия). Фолликулярная аденома по результатам гистологического исследования встречалась у 44,4% детей с ТУЗ при доброкачественных результатах тонкоигольной аспирационной биопсии (Bethesda II) и в 50% — при выявлении фолликулярной неоплазии (Bethesda IV).ЗАКЛЮЧЕНИЕ. Впервые в Российской Федерации проведен сравнительный анализ характеристик цитологического и гистологического исследования у детей с ТУЗ. Показательно, что только в 10,5% (n=2) случаев соответствовали цитологические и морфологические результаты. Выбор тактики радикального лечения должен учитывать высокую частоту несовпадений гистологических и морфологических исследований.

## Abstract (en)

BACKGROUND: Toxic nodular goiter (TNG) is a rare disease in which the cause of hyperthyroidism is the presence of a node or nodes that autonomously secrete thyroid hormones. With children and adolescents this condition is extremely rare — in 5–7.5% of all cases of nodular goiter. Therapy of toxic nodular goiter is aimed at relieving the symptoms of hyperthyroidism taking into account the malignant potential of the nodular formation. In the available literature, there are no data on the clinical course, comparative results of cytological and histological data in patients with toxic nodular goiter, which debuted in their childhood.AIM: Analysis of the features of the clinical course, comparison of the results of cytological and histological studies of toxic nodular goiter in children and adolescents.MATERIALS AND METHODS: A retrospective, single-center study of 21 patients with single-nodular toxic goiter, hospitalized at the Endocrinology Research Centre in the period from January 2016 to December 2019.RESULTS: The mean age at the time of the survey was 13.9 years. Thirteen patients (65%) had manifest thyrotoxicosis, and seven (35%) had subclinical hyperthyroidism. More than half of children — 57.1% (n = 12) did not receive thyreostatic therapy. The cytological picture in 11 patients (61.1%) corresponded to benign changes (nodular colloid goiter or adenomatous goiter) — Bethesda II, in 4 patients — follicular tumor — Bethesda IV, in 4 children the study was not informative. 19 patients (90.5%) underwent surgical treatment (hemithyroidectomy). According to the results of histological examination, follicular adenoma was found in 44.4% of children with nodular toxic goiter with benign results of TAB (Bethesda II) and was found in 50% with revealing follicular neoplasia (Bethesda IV).CONCLUSION: For the first time in the Russian Federation was carried out a comparative analysis of the characteristics of cytological and histological studies in children with toxic nodular goiter. It is significant that only in 10.5% (n=2) cytological and morphological results were consistent. The choice of radical treatment tactics should take into account the high frequency of mismatches between histological and morphological studies.

## ОБОСНОВАНИЕ

Токсический узловой зоб (ТУЗ) характеризуется появлением в щитовидной железе (ЩЖ) автономно функционирующих тиреоцитов, что является фактором риска развития тиреотоксикоза [1]. ТУЗ как причина тиреотоксикоза впервые описан в 1913 г. американским врачом Генри Стэнли Пламмером, который выделил два варианта токсического зоба: «экзофтальмический» и «аденоматозный». В 23% случаев «аденоматозный зоб» был ассоциирован с гипертиреозом и не сопровождался развитием экзофтальма [2].

ТУЗ преимущественно встречается среди взрослого населения (чаще у лиц старше 60 лет), до 10% среди всех одноузловых образований [1], тогда как в молодом возрасте встречаемость значительно реже — 5–7,5% [3].

С наибольшей частотой ТУЗ встречается в йододефицитных регионах: среди всех причин тиреотоксикоза многоузловой токсический зоб (МТЗ) встречается в 58% случаев, а одноузловой токсический зоб — в 10% [1]. Напротив, в йодообеспеченных регионах МТЗ выявляется всего в 15–30% всех случаев тиреотоксикоза [4].

Дифференцировать диффузно-токсический зоб и ТУЗ позволяет сцинтиграфия ЩЖ, при которой активно функционирующий узел («горячий узел») накапливает радиофармпрепарат (РФП), а окружающая тиреоидная ткань находится в состоянии супрессии (рис. 1).

**Figure:**
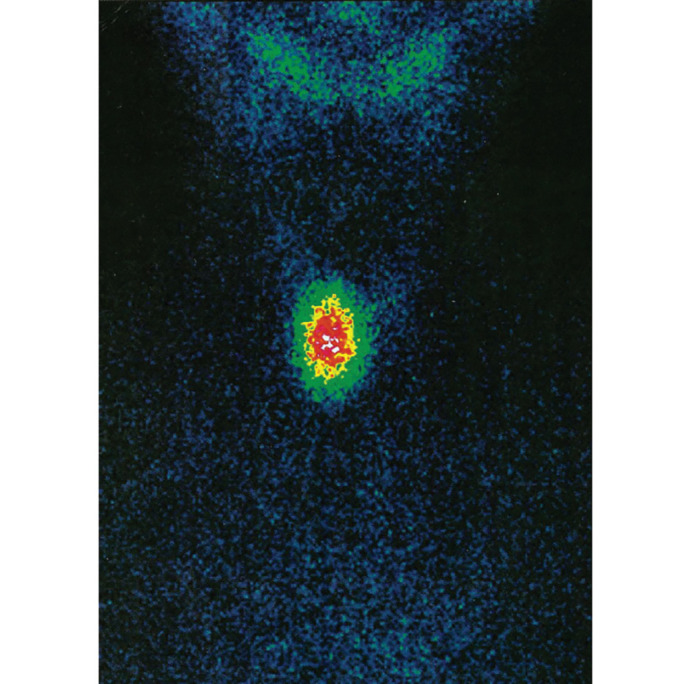
Рисунок 1. Активно функционирующий узел («горячий узел») по данным сцинтиграфии щитовидной железы с Тс-99м-пертехнетатом у пациента 13 лет с токсическим узловым зобом (собственное наблюдение).

В некоторых случаях автономия может носить диффузный характер за счет диссеминации автономно функционирующих участков в ЩЖ. Дополнительным критерием дифференциальной диагностики является отсутствие в сыворотке крови аутоантител к рецепторам тиреотропного гормона (АТрТТГ) при ТУЗ, в отличие от диффузно-токсического зоба (болезни Грейвса).

В настоящий момент существуют два типа радикального лечения ТУЗ — оперативное лечение и радиойодтерапия (РЙТ). Выбор тактики лечения зависит от многих причин, в том числе обусловлен возможными рисками анестезиологических пособий в случае хирургического лечения и развития гипотиреоза после проведения РЙТ [5]. У детей РЙТ при ТУЗ проводится редко в связи с потенциальной возможностью возникновения в отдаленном периоде рака ЩЖ [6]. Риск малигнизации узловых образований ЩЖ у детей может достигать 20% [5], аналогичная тенденция возможна и в случае автономно функционирующих образований [7–10].

В связи с крайне редкой встречаемостью ТУЗ ЩЖ у детей важно обобщить клинические данные детей с этим заболеванием.

## ЦЕЛЬ ИССЛЕДОВАНИЯ

Целью данного исследования является анализ особенностей клинического течения, сравнение результатов цитологического и гистологического исследований ТУЗ у детей и подростков.

## МАТЕРИАЛЫ И МЕТОДЫ

Место и время проведения исследования

Место проведения. Обследование и лечение пациентов проводилось в Институте детской эндокринологии ФГБУ «НМИЦ эндокринологии» Минздрава России.

Время исследования. В исследование включены пациенты, находившиеся в детской клинике с января 2016 г. по декабрь 2019 г.

Изучаемые популяции (одна или несколько)

Критериями включения в исследование являлись: возраст от 0 до 18 лет, наличие узлового зоба, наличие «горячего узла» по результатам сцинтиграфии, отрицательный титр АТрТТГ.

Критерии исключения: наличие диффузно-токсического зоба (болезни Грейвса), наличие узлового нетоксического зоба.

Дизайн исследования

Ретроспективное одноцентровое исследование, включающее в себя 21 пациента с одноузловым токсическим зобом. Дети проживали в различных регионах России (г. Москва — 2, по 3 ребенка из Московской и Ярославской областей, по 2 — из Тульской и Белгородской областей и по 1 ребенку из Калужской, Тверской, Рязанской, Курганской, Липецкой, Курганской областей, республик Удмуртии, Чувашии и Татарстана). Все перечисленные регионы являются регионами с легким йодным дефицитом [11–13]. В некоторых статьях авторы описывают два или более различных фрагмента исследования, которые выполняются с разными целями (что, вообще говоря, нежелательно в рамках одной статьи). В этом случае необходимо отдельно описывать дизайн каждого из фрагментов исследования.

Описание медицинского вмешательства

Всем пациентам было проведено комплексное клинико-гормональное и инструментальное обследование, включавшее в себя: сбор анамнеза жизни и заболевания, проведение ультразвукового исследования ЩЖ, определение содержания в сыворотке крови тиреотропного гормона (ТТГ), свободного тироксина (св. Т4), свободного трийодтиронина (св.Т3) и АТрТТГ, проведение тонкоигольной пункционной биопсии (ТАБ) под ультразвуковым контролем и морфологическое исследование узлового образования до и после оперативного лечения.

Методы регистрации исходов

УЗИ ЩЖ проводилось на аппарате Тoshiba Aplio 500 линейным датчиком PLT-1204ВХ с диапазоном частот 7–18 МГц. Исследование выполнялось в стандартном положении пациента лежа на спине с запрокинутой головой и подложенным под плечи валиком. Сканирование ЩЖ осуществлялось в В-режиме и с применением режима цветового допплеровского картирования (ЦДК). Производилось измерение трех размеров обеих долей ЩЖ (длина, ширина и переднезадний размер), объем ЩЖ вычислялся по формуле J. Brunn (1981 г.):

[ширина правой доли (см) × длина правой доли (см) × толщина правой доли (см) + ширина левой доли (см) × длина левой доли (см) × толщина левой доли (см)] × 0,479.

Оценивались структура ЩЖ, степень эхогенности, васкуляризация, наличие узловых образований (заведующая отделением — к.м.н. Т.В. Солдатова).

Исследование гормонов в сыворотке крови проводилось в лаборатории гормонального анализа ФГБУ «НМИЦ эндокринологии» Минздрава России (заведующая лабораторией Л.В. Никанкина). Лабораторные исследования были выполнены на автоматическом иммунохемилюминесцентном анализаторе ARCHITECT i2000sr (ABBOTT).

С целью сравнения лабораторных данных, выполненных в ФГБУ «НМИЦ эндокринологии» Минздрава России и по месту жительства, проводился пересчет уровней ТТГ, св.Т4 и св.Т3 в пмоль/л с помощью калькулятора пересчета единиц измерения анализов: https://www.slimhauz.ru/stoimost/analizy/kalkulyator_analizov.

Сцинтиграфия ЩЖ проводилась в отделе радионуклидной диагностики и терапии, осуществлялась на гамма-камерах ОФЭКТ Discovery NM630 и ОФЭКТ-КТ Discovery NM/CT670 (руководитель отдела — д.м.н. П.О. Румянцев) ФГБУ «НМИЦ эндокринологии» Минздрава России с применением 99mTc-пертехнетата.

Необходимая для исследования доза РФП рассчитывалась индивидуально с помощью калькулятора вводимой активности PedDose в [МБк] и [мКи] (https://www.eanm.org/publications/dosage-calculator).

Сцинтиграфия проводилась через 15–20 мин после внутривенного введения РФП в положении пациента лежа на спине, детектор гамма-камеры располагался максимально близко над шеей. Время исследования 10 мин. Затем врачом-радиологом на рабочей станции Xeleris (GI) проводилась оценка функционального состояния ЩЖ визуально и с помощью рассчитываемого программой индекса захвата РФП ЩЖ.

ТАБ узловых образований ЩЖ проводилась под контролем УЗИ с использованием иглы для внутримышечных инъекций диаметром 0,7 мм в положении больного лежа на спине с запрокинутой головой (без применения анестезиологического пособия). Цитологическое исследование пунктатов, окрашенных по Маю–Грюнвальду–Гимзе, проводилось в отделе фундаментальной патоморфологии (заведующий — д.м.н. А.Ю. Абросимов). Заключение цитологических исследований проводилось в соответствии с критериями классификации цитопатологии ЩЖ Bethesda (2009, 2017) [14].

Гистологическое исследование ткани ЩЖ, полученной в результате оперативного лечения (19 пациентов), проводилось в отделении фундаментальной патоморфологии. Тканевые образцы фиксировали 10% раствором формалина в течение 24 ч и заключали в парафиновые блоки по стандартной методике. Из парафиновых блоков готовили срезы толщиной 5 мкм, которые депарафинировали, обезвоживали и окрашивали гематоксилином и эозином по стандартной методике. Просмотр цитологических и гистологических стеклопрепаратов осуществлялся на микроскопах Leica/Nikon/Zeiss с увеличением 1×10, 1×20, 1×40, 1×100.

Этическая экспертиза

Проведение данного исследования одобрено локальным этическим комитетом по этике ФГБУ «НМИЦ эндокринологии» МЗ РФ (протокол № 3 от 26.02.2020 г.).

Статистическая обработка

Размер выборки предварительно не рассчитывался. Статистическая обработка материала проводилась с использованием программ Microsoft Office Excel 2010 и статистического пакета STATISTIСA (StatSoft, США). При нормальном распределении количественного признака данные представлены в виде среднего значения и стандартной ошибки среднего: M±SEM, если не указано другого. При отличном от нормального распределении количественного признака данные представлены в виде медианы значения и его интерквартильного размаха: Me (25; 75 перцентили), если не указано другого.

Для сравнения двух групп по количественным признакам рассчитывался критерий Стьюдента для параметрических выборок, для непараметрических — применялся тест Манна–Уитни. Взаимосвязь между двумя показателями оценивалась с использованием корреляционного анализа методом Спирмена. Для всех статистических методов значение р<0,05 считалось статистически значимым

Нежелательные явления

В ходе исследования нежелательных явлений зафиксировано не было.

## РЕЗУЛЬТАТЫ

Объекты (участники) исследования

За четырехлетний период (2016–2019) в круглосуточном стационаре детской клиники ФГБУ «НМИЦ эндокринологии» Минздрава России были обследованы 166 детей с одноузловым зобом, у 12,6% из них (n=21) был диагностирован одноузловой токсический зоб.

Основные результаты исследования

В данной статье проанализированы данные 21 ребенка (5 мальчиков, 16 девочек) с одноузловым токсическим зобом. Возраст на момент обследования в ФГБУ «НМИЦ эндокринологии» Минздрава России составил от 6,9 до 17,9 года (13,9±2,9 года).

Средний возраст детей на момент манифестации заболевания составлял 12,9±2,7 года. Следует отметить, что более чем в половине случаев (52,4%, 11/21) узловой токсический зоб протекал без явных клинических симптомов, и узел в ЩЖ был выявлен «случайно» на профилактическом осмотре. Однако при углубленном анализе анамнестических данных у каждого третьего ребенка (36,4%, 4/11) имелись такие жалобы, как слабость (25%), повышенная утомляемость (25%), тахикардия (25%), раздражительность (50%), нарушения сна (50%). Родители остальных детей (n=10) активно обратились к педиатру с жалобами на увеличение «объема шеи» и деформацию ее контуров. В половине случаев (5/10) зоб был единственной жалобой, у другой половины имелись жалобы на слабость (54,5%), повышенную раздражительность (45,5%), плаксивость (18,2%), чувство нехватки воздуха (18,2%), нарушения сна (9%), ощущение сердцебиения (27,3%).

Таким образом, в дебюте заболевания у 52,4% пациентов (11/21) узловой зоб был выявлен при проведении профилактических осмотров, у 23,8% — (5/21) единственной жалобой при обращении к педиатру было «увеличение объема шеи», и только каждого четвертого ребенка (23,8% (5/21)) беспокоили не только «увеличение шеи», но и специфические для тиреотоксикоза жалобы. При гормональном обследовании по месту жительства у 38,1% детей (n=8) выявлен субклинический гипертиреоз, у большинства детей (61,9%, n=13) — манифестный тиреотоксикоз. Обращает на себя внимание тот факт, что у пациентов с манифестным тиреотоксикозом объем узловых образований был достоверно выше по сравнению с пациентами с субклиническим тиреотоксикозом. При отсутствии отличий уровней св.Т4 у пациентов с манифестным тиреотоксикозом достоверно повышены уровни св.Т3 и снижен уровень ТТГ, что диктует необходимость обязательного определения уровня св.Т3 у пациентов с узловыми образованиями ЩЖ (табл. 1).

**Table:** Таблица 1. Лабораторно-инструментальные данные пациентов при первичном обследовании по месту жительства

Тиреоидный статус	Возраст, лет	ТТГ, мМЕ/л (0,43–4,2)	св.Т4, пмоль/л (10,1–17,9)	св.Т3, пмоль/л (2,8–6,3)	АТрТТГ, МЕ/л (0–1,75)	Объем ЩЖ, см3	Объем узла, см3
Субклинический тиреотоксикоз (n=8)	12,8±1,5	0,07[ 0,04; 0,2 ]	12,7[ 10,8; 14,6 ]	4,2 [ 4,1; 4,6 ]	0,3	14,9 [ 9,9; 19,4 ]	2,59 [ 0,54; 4,5 ]
Манифестный тиреотоксикоз (n=13)	12,7±3,3	0,01 [ 0,008; 0,02 ]	19,7 [ 14,9; 27,0 ]	8,3 [ 7,1; 10,7 ]	0,3	17,0 [ 9,8; 25,1 ]	8,3 [ 4,7; 10,9 ]
р	р=0,93	р=0,015	p=0,09	р=0,0005		р=0,585	р=0,021

Корреляционный анализ выявил умеренную обратную корреляционную зависимость между объемом узла и уровнем св.Т3 (р=-0,39).

Таким образом, в дебюте заболевания у детей с ТУЗ степень выраженности тиреотоксикоза может быть различной, однако в случаях манифестного тиреотоксикоза объем узла был достоверно больше, чем у пациентов с субклиническим тиреотоксикозом.

Все дети в течение первого года после диагностики узлового зоба были направлены для дополнительного обследования в ФГБУ «НМИЦ эндокринологии» Минздрава России. Медиана стажа динамики наблюдения составила 0,34 года [ 0,2; 1,06 ]. Средний возраст детей (n=21) при поступлении в детскую клинику составлял 13,7±2,9 года. Более половины детей — 57,1% (n=12) не получали тиреостатической терапии, остальные дети (n=9) получали тиамазол (Ме 0,205 мг/кг [ 0,15; 0,31 ]). Медиана длительности терапии составила 1,5 мес [0,87; 2,63]. У пациентов на терапии достигнута клинико-лабораторная компенсация. Показатели гормонального статуса у пациентов без лечения в среднем соответствовали субклиническому тиреотоксикозу. Из 12 пациентов, не получавших лечение, у 8 пациентов сохранялся субклинический гипертиреоз, у 4 пациентов — манифестный тиреотоксикоз (табл. 2).

У 8 пациентов (38%) по результатам УЗИ ЩЖ выявлена повышенная васкуляризация в объемном образовании (рис. 2).

**Figure:**
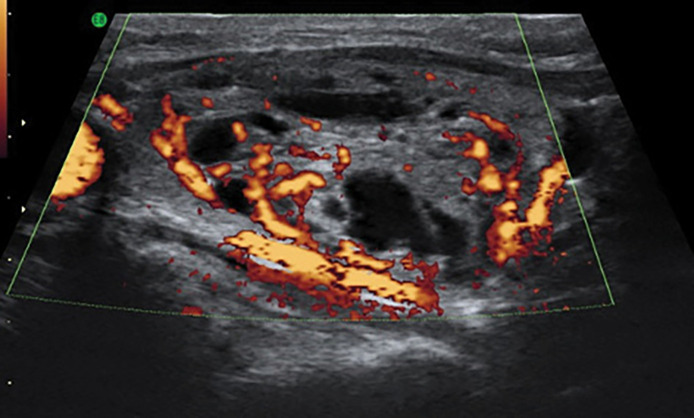
Рисунок 2. Продольное изображение узлового образования в режиме энергетического допплеровского картирования у пациента 12 лет с токсическим узловым зобом (собственное наблюдение).

С диагностической целью всем пациентам (n=21) была проведена сцинтиграфия ЩЖ, по результатам которой у всех пациентов был выявлен «горячий узел».

ТАБ была выполнена 19 из 21 детей: цитологическая картина у 9 пациентов (47,3%) соответствовала доброкачественным изменениями (узловой коллоидный зоб или аденоматозный зоб) — Bethesda II, у 4 пациентов (21,0%) — фолликулярной опухоли — Bethesda IV, у 3 детей исследование оказалось неинформативным. По техническим причинам ТАБ не была проведена 3 пациентам.

Двум пациентам проведена РЙТ; в обоих случаях цитологическая картина соответствовала Bethesda II. Объем ЩЖ составил 10,8 и 7,1 см3, а объем узловых образований — 4,5 и 2,4 см3 соответственно (TIRADS 2). В обоих случаях РЙТ была проведена в связи с небольшим размером узла и настойчивым желанием родителей.

19 пациентам (90,5%) было проведено хирургическое лечение (гемитиреоидэктомия): 18 пациентам в условиях ФГБУ «НМИЦ эндокринологии» Минздрава России, 1 пациенту по месту жительства. По результатам морфологического исследования послеоперационного материала у 11 детей (57,8%) диагностирован активно пролиферирующий коллоидный зоб (рис. 3a, b, c, 4), у 8 (42,1%) — фолликулярная аденома ЩЖ (рис. 5a, b, 6a, b).

**Figure:**
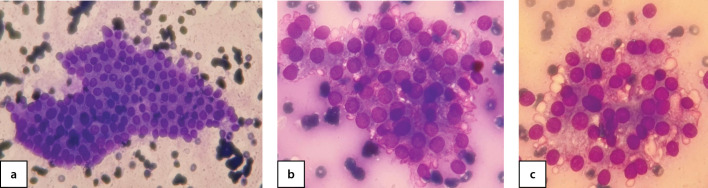
Рисунок 3 а, b, c. Цитограмма узлового коллоидного активно пролиферирующего зоба с формированием фолликулярных и трабекулярных структур и вакуолей резорбции коллоида (b, c), окраска азур-эозин, увеличение 40х (а), 100 х (b, c) (собственное наблюдение).

**Figure:**
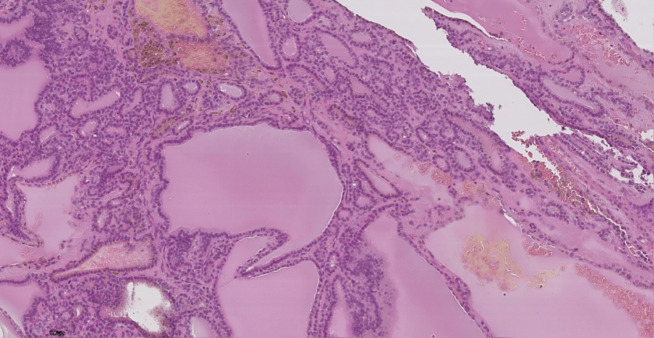
Рисунок 4. Узловой активно пролиферирующий зоб с варьированием формы и размера фолликулов, очагом кровоизлияния и скоплением гемосидерофагов, окраска гематоксилин-эозин, увеличение 20х (собственное наблюдение).

**Figure:**
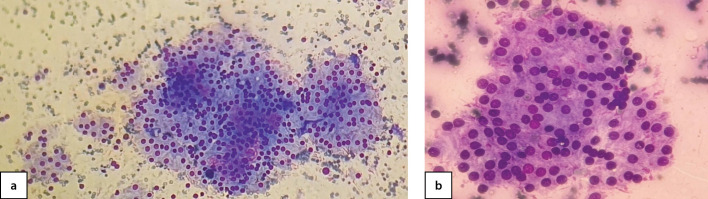
Рисунок 5 а, b. Цитограмма активно пролиферирующего (аденоматозного) зоба, окраска азур-эозин, увеличение 10х (а), 100х (b) (собственное наблюдение).

**Figure:**
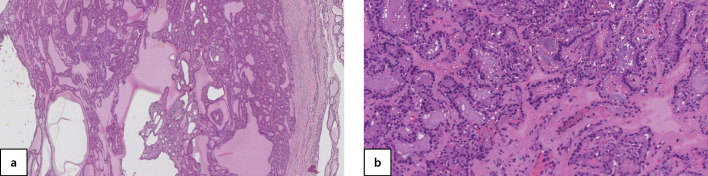
Рисунок 6 а, b. Узловой микрофолликулярный коллоидный зоб с очагами сосочковой пролиферации фолликулярного эпителия, окраска гематоксилин-эозин, увеличение 20х (a), 40х (b) (собственное наблюдение).

Обращает на себя внимание тот факт, что фолликулярная аденома выявлялась как у пациентов с Bethesda IV (2/4), так и у пациентов с установленным на дооперационном этапе доброкачественным заключением — Bethesda II (4/11), в 1 случае фолликулярная аденома обнаружена у пациента без предварительно проведенной ТАБ. У остальных пациентов гистологическая картина соответствовала активно пролиферирующему, коллоидному в разной степени зобу (табл. 3).

Таким образом, фолликулярная аденома по результатам гистологического исследования встречалась у 41,7% детей с ТУЗ при неинформативных и доброкачественных результатах ТАБ (Bethesda I–II) и в 50% случаев соответствовала выявленной на дооперационном этапе фолликулярной неоплазии (Bethesda IV).

Катамнестический мониторинг 14 пациентов показал, что у 10 пациентов в течение года после радикального лечения сохранялся эутиреоз, у 4 развился гипотиреоз, потребовавший назначения заместительной терапии.

**Table:** Таблица 2. Данные динамического наблюдения у пациентов в зависимости от терапии

	n	Возраст	ТТГ, мМЕ/л (0,43–4,2)	св.Т4, пмоль/л (10,1–17,9)	св.Т3, пмоль/л (2,8–6,3)	Объем ЩЖ, см3	Объем узла, см3
Тиреостатическая терапия	9	12,6±3,8	1,79 [ 0,034; 9,904 ]	10,4 [ 8,93; 11,2 ]	4,6 [ 3,96; 5,23 ]	18,7 [ 13,5; 28,3 ]	9,9 [ 7,2; 17,2 ]
Без терапии	12	14,8±1,9	0,02 [ 0,002; 0,394 ]	13,2 [ 10,87; 14,33 ]	6,1 [ 5,3; 7,1 ]	11,6 [ 10,6; 20,95 ]	6,8 [ 3,6; 12,5 ]
р		р=0,145	р=0,028	р=0,086	р=0,004	р=0,37	р=0,39

 

**Table:** Таблица 3. Соотношение цитологической и морфологической картины у детей с токсическим узловым зобом

Результаты ТАБ (n=18)	n	Результаты гистологии (n=19)
фолликулярная аденома	активно пролиферирующий зоб
Bethesda I	3	1	2
Bethesda II	9	4	5
Bethesda IV	4	2	2
Не проводилась	3	1	2
Всего	19	8	11

## ОБСУЖДЕНИЕ

Репрезентативность выборок

По литературным данным, распространенность ТУЗ у взрослых составляет 10–32% [1][15], у детей данные единичны [3].

В отечественной литературе понятия «токсический зоб» и «функциональная автономия щитовидной железы» часто являются синонимами [16]. В зарубежных источниках используется термин «токсический узловой зоб» [17]

ТУЗ у детей в дебюте заболевания может протекать как с жалобами, подозрительными в отношении тиреотоксикоза, так и без них (субклинический тиреотоксикоз).

В нашей группе у 76,2% детей основным клиническим симптомом было увеличение «объема шеи», специфические жалобы отмечались у 42,9% пациентов. При обследовании у 61,9% детей — повышение хотя бы одного из двух тиреоидных гормонов. Отрицательный титр АТрТТГ и данные сцинтиграфии ЩЖ позволили достоверно дифференцировать аутоиммунный и деструктивный тиреотоксикоз у всех обследуемых нами пациентов.

Отечественные авторы определяют следующие стадии функциональной автономии ЩЖ: компенсированная (эутиреоз), субкомпенсированная (субклинический гипертиреоз) и декомпенсированная (манифестный гипертиреоз) [1].

В серии описанных нами случаев на момент постановки диагноза 38,1% пациентов — в состоянии субклинического тиреотоксикоза, 61,9% — в состоянии манифестного тиреотоксикоза. У всех пациентов отмечался отрицательный титр АТрТТГ.

Клиническая значимость результатов

Единственным дооперационным методом оценки структурных изменений и установления цитологических параметров образований в ЩЖ является ТАБ, особенно при размерах узловых образований более 1 см. ТАБ является обязательной в первую очередь для оценки злокачественного потенциала узлового образования ЩЖ.

Цитологическое исследование пунктата узлового образования ЩЖ не позволяет надежно дифференцировать доброкачественную опухоль — фолликулярную аденому — от рака ЩЖ. Достоверно диагностировать потенциал злокачественности новообразований возможно только на основании послеоперационного гистологического исследования. У взрослых пациентов результаты ТАБ, свидетельствующие о фолликулярной неоплазии или подозрении на фолликулярную неоплазию (Bethesda IV), в 15–30% случаев в конечном итоге оказываются злокачественными образованиями [18], в детской практике описано до 58% злокачественных новообразований [19][20].

В большинстве случаев ТУЗ — это доброкачественное образование с низким риском малигнизации [9]. Однако в связи с риском более агрессивного течения рака ЩЖ в детском возрасте гистологическое исследование помогает обнаружить случайную опухоль, расположенную в гиперфункционирующем узле. J.J. Smith и соавт. [21] и Y. Senyurek Giles [22] показали, что при токсическом одноузловом зобе у взрослых в 12–18,3% случаев узлы оказались злокачественными. M. Niedziela и соавт. [15] оценили риск малигнизации при ТУЗ у детей (n=31), злокачественный процесс обнаружен в 29% случаев. По данным исследования Croom и соавт. [23], у детей и подростков риск малигнизации при ТУЗ отмечался в 11,3%.

В нашей серии наблюдений прооперировано 19 детей (гемитиреоидэктомия), ни в одном случае злокачественных образований и фолликулярных опухолей неопределенного риска злокачественности выявлено не было. При этом медиана стажа наблюдения от момента манифестации до момента радикального лечения — 0,34 года [ 0,2; 1,06 ].

По результатам нашей работы совпадение цитологического и гистологического результатов наблюдалось у 60% пациентов: при Bethesda II — в 55,6%, при Bethesda  IV— 50%.

Следует отметить, что в нашей работе у каждого третьего ребенка (36,4%) при цитологическом заключении Bethesda I–II результаты послеоперационной гистологии свидетельствовали о фолликулярной аденоме (у 4 из 11 детей).

Несмотря на проведение гемитиреоидэктомии, после проведения оперативного лечения в течение последующих 3 мес у 15,7% пациентов развился послеоперационный гипотиреоз, потребовавший назначения заместительной терапии. После проведения РЙТ у 1 пациента сохраняется эутиреоз, второму ребенку потребовалось назначение заместительной гормональной терапии.

## ЗАКЛЮЧЕНИЕ

ТУЗ в детском возрасте — редкое заболевание. Консервативная терапия при данном заболевании направлена на купирование симптомов гипертиреоза.

Полученные нами на небольшой группе пациентов данные только в 10,5% случаев (2 пациента) выявили соответствие результатов цитологических и морфологических результатов.

Ни в одном случае не было обнаружено злокачественное образование или образование неопределенного риска злокачественности. Данное обследование было проведено у пациентов детского возраста с малым стажем динамического наблюдения.

РЙТ может быть рассмотрена в качестве радикального метода лечения ТУЗ у детей и подростков при отсутствии признаков злокачественного роста узлового образования.
